# The Role of MicroRNAs in Development and Function of Regulatory T Cells – Lessons for a Better Understanding of MicroRNA Biology

**DOI:** 10.3389/fimmu.2020.02185

**Published:** 2020-09-09

**Authors:** Heike Kunze-Schumacher, Andreas Krueger

**Affiliations:** Institute for Molecular Medicine, Goethe University Frankfurt am Main, Frankfurt am Main, Germany

**Keywords:** miRNA, regulatory T cell, Treg cell, thymus, T-cell receptor, gene regulation

## Abstract

MicroRNAs (miRNAs) have emerged as critical posttranscriptional regulators of the immune system, including function and development of regulatory T (Treg) cells. Although this critical role has been firmly demonstrated through genetic models, key mechanisms of miRNA function *in vivo* remain elusive. Here, we review the role of miRNAs in Treg cell development and function. In particular, we focus on the question what the study of miRNAs in this context reveals about miRNA biology in general, including context-dependent function and the role of individual targets vs. complex co-targeting networks. In addition, we highlight potential technical pitfalls and state-of-the-art approaches to improve the mechanistic understanding of miRNA biology in a physiological context.

## Introduction

Regulatory T (Treg) cells are critical to maintain tolerance against self-antigens as well as innocuous antigens, such as commensal bacteria. Loss of Treg cells or their function results in fatal autoimmune disease in mice and humans. Treg cells express the lineage-defining transcription factor Foxp3, which controls transcriptional programs critical for Treg-cell function [for review see (1)].

Treg cells emerge via two distinct mechanisms. Thymus-derived (t)Treg cells are generated in the thymus as a stable lineage, dependent on strong T-cell receptor (TCR) signals. Induced or peripheral (i)Treg cells are generated in the periphery from naive precursors. In the thymus, tTreg cells are formed through either one of two intermediate precursor populations, characterized by mutually exclusive expression of Foxp3 and CD25, before mature Treg cells become Foxp3^+^CD25^+^ double-positive (2–4). The underlying molecular mechanisms promoting Treg-cell generation through one or the other intermediate remain partially elusive. However, it has been established that CD25^+^ precursors can be selected on self-antigen and have encountered stronger TCR signals than their mature CD25^+^Foxp3^+^ progeny (5, 6). In addition, its two-step development via a CD25^+^ intermediate relies on a sequence of TCR signaling to generate the intermediate followed by IL-2 signals to ultimately consolidate Treg-cell fate (2, 3). Foxp3^+^ precursors are partially dependent on cytokine-driven signals, such as via IL-15 for development and require weaker TCR signals (7, 8). As a consequence, both Treg cell precursor populations have distinct TCR repertoires (8). Notably, it has been suggested that tTreg cells generated through different pathways display distinct functions in the periphery (8).

A multitude of mechanisms of Treg-cell mediated immunosuppression have been described and Treg cells may employ such mechanisms non-exclusively in different contexts [for review see (9, 10)]. Treg cells release immunomodulatory cytokines, including IL-10 and TGFβ. They are able to interfere with cellular metabolism, for instance through release of IDO and consequent depletion of tryptophan, or via cAMP (11). It has also been suggested that Treg cells capture IL-2, the prototypic pro-proliferative T-cell cytokine. Furthermore, Treg cells constitutively express high levels of CTLA-4. CTLA-4 is an antagonistic receptor for the co-stimulatory ligands CD80 and CD86. Treg-cell specific deletion of *Ctla4* has revealed a critical role of CTLA-4 in Treg-cell specific function at steady-state: Loss of CTLA-4 in Treg cells results in fatal autoimmune disease, largely phenocopying total Treg-cell deficiency and suggesting that CTLA-4 is critical in maintaining immunological tolerance of a polyclonal T-cell repertoire (12). Mechanistically, it has been demonstrated that CTLA-4 acts predominantly by scavenging CD80 and CD86 from the surface of antigen-presenting cells thus preventing the transmission of co-stimulatory signals (13, 14).

MicroRNAs (miRNAs) are short non-coding RNAs of 18–23 nucleotides in length. They are generated from primary transcripts through a series of processing steps. In mammals, the majority of pri-miRNAs are transcribed as non-protein coding transcripts driven by polymerase II promoters. In addition, a small number of pri-miRNAs, termed miRtrons, are generated through splicing. Pri-miRNAs are cleaved by an endonuclease complex containing Drosha and Dgcr8 to generate pre-miRNA hairpins, which are exported from the nucleus by exportin 5 for further processing. The endonuclease Dicer removes the hairpins thus releasing the mature miRNAs, the more thermodynamically stable strand of which is then loaded into the RNA-induced silencing complex (RISC). In addition to the miRNA, the RISC consists of Argonaute family proteins as well as GW182. Targeting of the miRNA-loaded RISC complex to an mRNA is mostly mediated by complementarity of nucleotides 2–7 of the miRNA, termed seed region. However, extended complementarity around the seed region increased targeting efficiency to a miRNA recognition element (MRE), and additional complementary regions as well as multiplicity of MREs, proximity of multiple MREs and sequence context might also contribute to targeting efficiency (15, 16). Prototypical functional MREs are predominantly located in the 3′ untranslated region (UTR) of an mRNA, although MREs, mostly favoring non-canonical miRNA interactions, are also found in the 5′UTR or the coding sequence of an mRNA. MiRNA binding to an mRNA target predominantly results in repression, with few exceptions also showing positive gene regulatory effects (17). MiRNA-mediated repression occurs by both mRNA destabilization followed by degradation as well as translational repression. It has been suggested that, at least in mammalian cell lines, the former mechanism is predominant (18). However, both mechanisms may also be kinetically linked (19).

## miRNA-Mediated Control of Treg-Cell Development

**FIGURE 1:**
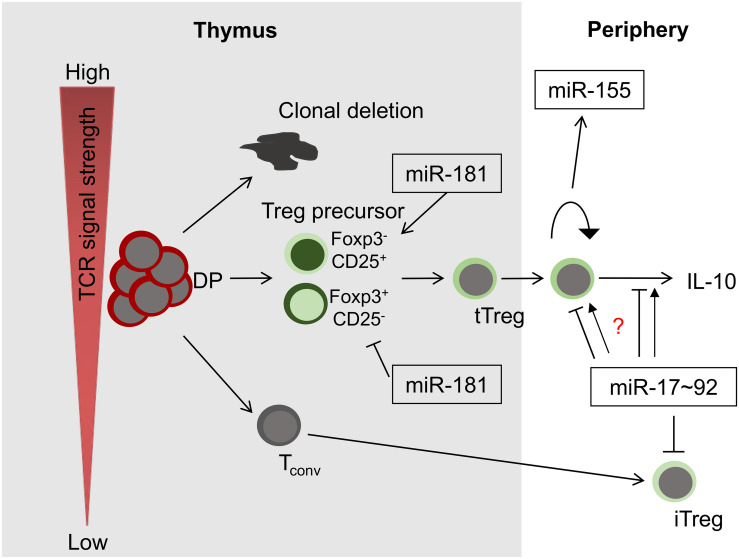
miRNAs in Treg-cell development and function. Among others, three miRNAs play critical roles in these processes, miR-181a/b-1, miR-155 and miR-17∼92. Whereas miR-181 exerts its function predominantly during intrathymic tTreg-development, miR-155 and miR-17∼92 control Treg-cell homeostasis and function. Of note, the role of miR-17∼92 promoting or limiting Treg-cell function remains unclear (indicated by a question mark).

Initial evidence for a critical role of post-transcriptional control of Treg cells by miRNA was obtained from mice with T-cell specific deficiency in the miRNA processing machinery. Thus, intrathymic Treg-cell development was impaired in such mice and they acquired inflammatory pathology (20). Treg-cell specific deletion of Dicer results in fatal autoimmune disease in mice due to a defect in Treg-cell homeostasis, loss of suppressive function as well as lineage stability (21, 22). Interestingly, Treg-cell derived miRNAs delivered via exosomes have been implicated as mediators of suppression (23). At present, no single miRNA has been identified, deletion of which fully mimics pan-miRNA deficiency. Thus, Treg-cell development and function are most likely controlled by multiple miRNAs, which might act in concert or in an isolated manner. Focusing mostly on evidence obtained from mouse models, we summarize the functions of some critical miRNAs (Figure 1). Rather than providing a comprehensive overview of miRNAs contributing to Treg-cell development and function, which can be found elsewhere (24–26), we focus on a set of miRNAs that may serve as examples for fundamental principles of miRNA biology. Thus, we highlight Treg cells as a model system to study miRNA function *in vivo*.

### MiR-155

MiR-155 is widely expressed in hematopoietic cells and its deletion results in defects in B-cell memory formation and a diminished germinal center reaction (27, 28). Moreover, loss of miR-155 reduces dendritic cell function and skews T cells toward the Th2 lineage (29). Absence of miR-155 in T cells conferred resistance to experimental autoimmune encephalitis (EAE), a mouse model resembling multiple sclerosis, supporting a pro-inflammatory role of miR-155 (30). Notably, Foxp3 positively regulates expression of miR-155 and deletion of miR-155 results in reduced Treg-cell numbers in thymus and periphery of mice (31, 32). Analysis of a Treg-cell intrinsic function revealed that miR-155 was required to maintain Treg-cell homeostasis and competitive fitness of Treg cells over time, but did not affect Treg cells’ suppressive capacity (31, 32). Loss of Treg-cell fitness was associated with reduced responsiveness to IL-2. Consistently, the negative regulator of cytokine signaling SOCS1 was identified as a target of miR-155 and transgenic expression of SOCS1 partially phenocopied loss of miR-155, whereas deletion of SOCS1 had the opposite effect (31). Given that an individual miRNA has a multitude of putative mRNA targets, a genetic phenocopy provides only indirect, but no stringent mechanistic, evidence for a physiologically relevant miRNA-mRNA target relationship. In order to address this issue directly, mice were engineered carrying alleles of SOCS1 specifically disrupting the interaction of *Socs1* mRNA with miR-155 (33). Analysis of these mice revealed that the reduction of Treg-cell numbers observed in miR-155-deficient mice were not attributable to aberrant upregulation of SOCS1. Conversely, Treg-cell fitness was directly dependent on a miR-155/SOCS1 targeting relationship. Similar selective effects were also observed for the role of the miR-155/SOCS1 axis during an antiviral response. This type of experiment currently represents the gold standard for analysis of miRNA-mRNA target relationships.

### MiR-181

MiR-181 is a family comprised of six miRNAs, encoded in clusters of two family members each, miR-181a/b-1, miR-181a/b-2, and miR-181c/d. MiR-181a/b-1 is among the most abundant miRNAs in thymocytes with its expression reaching a distinct peak in double-positive cells followed by a sharp decline (34, 35). Modulation of expression of miR-181a in thymocytes resulted in alterations of TCR signaling and suggested a function in positive selection, consistent with its expression pattern (36, 37). Notably, mice deficient in miR-181a/b-1 displayed defects in selection that were most prominent in unconventional T cells, such as NKT and MAIT cells, but not γδT cells (38–42). However, the consequences of loss of miR-181a/b-1 in deletion of conventional T cells were more complex, precluding simple conclusions with regard to miR-181a’s role in positive or negative selection (40). Loss of miR-181a/b-1 also resulted in inefficient *de novo* generation of tTreg cells (43). Interestingly, Foxp3^+^ and CD25^+^ developmental intermediates were differentially affected by loss of miR-181a/b-1. Foxp3^+^ precursor frequencies were decreased proportionally to the impaired formation of mature Treg cells, whereas, surprisingly, frequencies of CD25^+^ precursors were increased, but could not compensate the defect in Treg-cell generation. Surprisingly, miR-181a/b-1-deficient Treg cells expressed elevated levels of CTLA-4 through a post-transcriptional, miR-181a/b-1-independent, mechanism, which resulted in elevated suppressive capacity *in vivo* (43). The mechanisms of how miR-181a/b-1 affects T-cell development remain only partially explored. It has been shown that miR-181a targets multiple phosphatases, which display negative regulatory functions in TCR signaling, including Ptpn22, SHP-2, Dusp5, and Dusp6 (36). Consistently, loss of miR-181a/b-1 dampens TCR signaling (38, 40). Interestingly, only silencing a combination of phosphatases was able to mimic the effect of depletion of miR-181a on TCR signaling, highlighting a potential critical role of miRNAs in simultaneously targeting multiple components of the same cellular pathway (36). Rescue experiments to restore development of NKT cells, MAIT cells, and Treg cells through increasing TCR signal strength suggest that in developing T cells the interaction of miR-181a and its phosphatase targets constitutes the most plausible molecular mechanism (38, 39, 42, 43). Thus, it is conceivable that, in the absence of miR-181a/b-1, TCR signals are insufficient to produce Foxp3^+^ precursors, but in turn limit TCR signal strength sufficiently to rescue some CD25^+^ precursors from clonal deletion. Nevertheless, additional targets may also contribute to miR-181a’s role as regulator of T-cell development and function. It has been suggested that miR-181 acts as a metabolic rheostat regulating expression of Pten (44). In addition, Bcl-2 family members might contribute to miR-181a-dependent survival of thymocytes and T cells upon stimulation (34, 45). Finally, regulation of thymic egress through modulation of S1PR1 may also affect peripheral T-cell function and tolerance (40). In the context of multiple plausible candidates, it remains a big challenge to identify potentially functionally relevant targets, especially if targets function coordinately rather than independently from each other.

### MiR-17∼92

MiR-17∼92 is a cluster of six miRNAs of related families, which plays critical roles in lung development, B-cell development, pre-thymic, and intrathymic T-cell development as well as Tfh cell formation (46–48). In Treg cells the role of miR-17∼92 remains controversial. Mice lacking miR-17∼92 in all T cells show increased resistance to EAE (49). Consistently, Treg cells from these mice produced more IL-10 and displayed elevated suppressive capacity (49, 50). In turn, overexpression of miR-17 resulted in elevated levels of IL-17 (49). Mechanistically, it has been suggested that miR-17 targeted Eos, a co-factor of Foxp3 in Treg cells, thus restricting the core Treg-cell lineage transcriptional program (49). In addition, it has been suggested that miR-17 restricted formation of iTreg cells by targeting TGFβ-RII and Creb1 (50). Surprisingly, deletion of miR-17∼92 specifically in Treg cells alone resulted in virtually opposite effects (51). Here, loss of miR-17∼92 increased susceptibility to EAE due to selective loss of antigen-specific Treg cells and possibly due to reduced expression of IL-10. The underlying reasons for these discordant findings remain unclear, but might in part be due to the fact that loss and overexpression of miR-17∼92 affects conventional T cells as well (50).

## Lessons for miRNA Biology in General

### Pan-miRNA Deficiency vs. Individual miRNAs

So far, in Treg cells no individual miRNA has been described, deletion of which phenocopies loss of virtually all miRNAs through deletion of the miRNA processing machinery. A simple, but unlikely, explanation might be that such dominant miRNA entity has not been discovered yet in Treg-cell biology, whereas they exist for other cell types, including MAIT cells, which are critically dependent on miR-181a/b-1 to a similar extent as on all miRNAs (39). Identifying groups of unrelated miRNAs that might act in concert to determine Treg-cell function might be difficult to identify using classical genetic tools. However, reconstitution of selected pre-miRNAs or even libraries in Drosha or Dgcr8-deficient cells might constitute a feasible approach (52). MiRNA-independent functions of the miRNA processing machinery might constitute an alternative explanation. Although not demonstrated in Treg cells, phenotypic differences between deletions of distinct components of miRNA processing, such as Dicer and Drosha, support such a possibility (53). Finally, it has been suggested that miRNAs may play a more general rather than gene-specific role in regulation of gene expression by limiting protein expression noise (54–56). Although not directly demonstrated in the context of primary cells, miRNAs may prevent spikes in mRNA levels through post-transcriptional degradation thus promoting narrow windows of protein expression. It remains to be shown, if and to what extent excessive fluctuations in protein expression might compromise Treg cells.

### “Master Targets” vs. Coordinated mRNA Targeting vs. Indirect Effects

**FIGURE 2:**
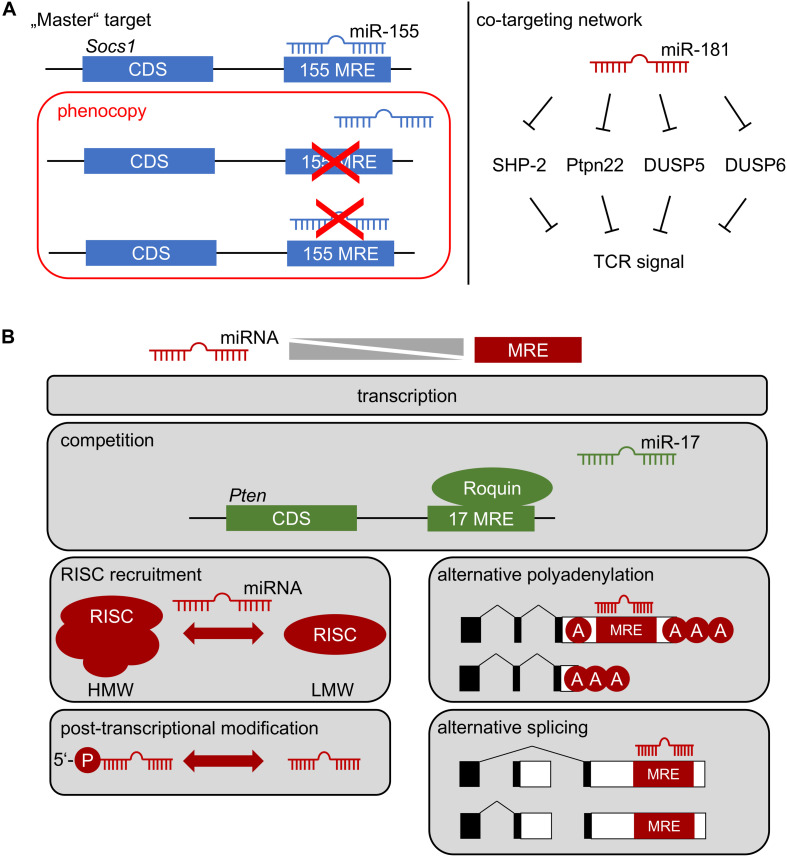
**(A)** How a miRNA affects cellular function: Left, repression of one critical mRNA by an individual miRNA determines cell function. Deletion of specific miRNA recognition elements (MREs) through genetic engineering constitutes the gold-standard to identify “master” targets. The miR-155/SOCS1 axis represents such an example in Treg-cell biology. Right: co-targeting networks. A single miRNA might target multiple mRNAs within the same pathway, generating functional outputs despite minor regulatory effects at the level of individual targets. Repression of multiple tyrosine and dual-specificity phosphatases within the TCR signaling pathway by miR-181 may represent such a scenario. Note, that in both cases multiple alternative targets of miR-155 and miR-181 have been identified, which are likely to contribute to cellular function. **(B)** Context-dependent miRNA function. The function of a miRNA most likely depends on the balance between its effective concentration and the concentration of accessible MREs. This balance is determined by miRNA and mRNA transcription, possible competition of access to MREs, selective recruitment to functional RISC complexes (high molecular weight, HMW, vs. low molecular weight, LMW), post-transcriptional regulation of miRNA, as well as alternative polyadenylation and splicing of mRNA.

Few examples of gold-standard experiments exist, in which one mRNA was unambiguously assigned a downstream role of miRNA-mediated control as a bona fide “master target.” The miR-155/SOCS1 axis constitutes one of these examples (Figure 2A) (33). However, as indicated above, it has become evident from specific deletion of the miR-155 MRE in *Socs1*, that only some functions were mechanistically dependent on this interaction. Thus, whereas Treg-cell fitness and NK-cell function in anti-viral immunity are dependent on this axis, many additional phenotypes of miR-155 deficiency were not, although functional relevance of SOCS1 dysregulation would have been plausible there as well (33). Two major mRNA targets, Pten and the pro-apoptotic Bcl-2 family member Bim have been at the center of functional investigation of miR-17∼92. Notably, neither heterozygous nor homozygous loss of Pten rescued the effects of miR-17∼92-deficiency in Treg cells (51). Furthermore, disruption of miR-17∼92 MREs in *Bim* had almost no effect on B-cell development, despite a prominent role of Bim in apoptosis of B-lineage cells (57–59). In contrast, lung development was exclusively dependent on the miR-17∼92/Bim axis (58). These experiments highlight the likely sparseness of “master targets,” although their existence might be partially obscured by the time consuming and almost exceedingly vast experimental effort, given that each miRNA has hundreds of theoretically predicted targets. Most likely at least a handful experimentally validated and plausible targets remain to be genetically engineered for any given miRNA. Moreover, it has been suggested that miRNAs act efficiently by moderately regulating multiple components of a given cellular pathway (60). The role of miR-181a/b-1 as a rheostat of TCR signaling may constitute such an example (Figure 2A) (36). However, experimental validation of co-targeting networks, such as TCR-signal dampening phosphatases, is currently not accessible to the gold-standard approach described above, because it would require simultaneous disruption of multiple MREs in different mRNAs. Improvements in CRISPR/Cas9-mediated gene editing may pave the way for such experiments. Understanding the role of individual miRNAs targeting distinct mRNAs is further complicated by the complexity of post-transcriptional gene regulation also including RNA-binding proteins (RBPs). Whereas miRNA-based mRNA targeting based on primary sequence defined MREs is comparatively well understood, binding of RBPs to 3′UTRs in many cases does not depend on defined sequence motifs, but rather on secondary structure (61, 62). Therefore, it remains challenging to predict potential cooperative or competitive action between RBPs and miRNAs. Recently, it has been demonstrated that the RBP Roquin competed with binding of miR-17∼92 to the 3′UTR of Pten (Figure 2B). In the absence of Roquin family members in Treg cells, miR-17∼92 was able to post-transcriptionally repress Pten, resulting in aberrant activation of the mTOR pathway and promotion of a T follicular regulatory cell phenotype (63). Thus, in Treg cells Roquin at least in part prevents autoimmunity by outcompeting miR-17∼92 binding to the Pten 3′UTR. Given the ill-defined nature of RBP binding requirements to 3′UTRs, the consequences of “gold-standard” deletion of MREs to characterize miRNA targets remain difficult to predict. Competition or cooperativity of miRNAs with RBPs might also contribute to explaining context-dependent miRNA function.

### Context-Dependent miRNA Function

The rules of hierarchical targeting of MREs by a specific miRNA remain ill-understood. Biochemical analyses and studies in cell lines have provided an extensive set of targeting rules based on composition of an MRE, its sequence context and cooperativity (64, 65). However, it remains an open question, whether the same targeting rules apply *in vivo* as well. It is plausible that quantitative relationships play a critical role in miRNA-mediated repression. Thus, a large concentration of candidate MREs in a cell may generate competition between targets and favor repression of targets with high affinity or multiple MREs (Figure 2B). Such a scenario has been exploited experimentally to functionally deplete miRNAs by ectopically expressing so-called “sponges” (66, 67). Conversely, based on quantitative estimates a regulatory function of competing endogenous RNAs has been questioned (68). Concentration-dependent function of miRNA-mediated gene repression *in vivo* has been directly demonstrated in developing B cells expressing an allelic series of miR-17∼92, including deficient cells, wild-type cells, and cells with mild over-expression of miR-17∼92 (69). This study identified virtually non-overlapping gene sets differentially affected by loss or over-expression of miR-17∼92, suggesting that already mild differences in expression may have a critical impact on a miRNA’s targetome. A comprehensive study comparing targetomes of miR-155 in four different hematopoietic cell types revealed that cell-context dependent repression was unlikely to be mediated by endogenous “sponges,” alternative polyadenylation and, thus, shortening of 3′UTRs, and alternative splicing (70). Notably, this study suggested that canonical MREs were more indicative of cell-type independent function, whereas non-canonical MREs were preferentially targeted in a context-dependent manner. Another striking example of context-dependent function is miR-21. It has been proposed that this miRNA is selectively functional mostly in transformed cells, whereas even high expression of this miRNA does not result in repression of predicted targets in primary cells (71). Consistently, despite prominent and dynamically regulated expression, miR-21 was completely dispensable for T-cell development, including Treg cells (72). As described for other cases of context-dependency, the mechanisms underlying the lack of miR-21 function in T-cell development remain completely elusive. Post-transcriptional regulation of miRNAs might contribute to this scenario (Figure 2B). It has been shown that only 5′-phosphorylated miR-34 was associated with RISC complexes and that 5′-phosphorylation was subject to direct regulation in response to DNA damage (73). Selective RISC-loading and assembly of functional high molecular weight (HMW) RISC complexes has also been proposed as a mechanism to regulate miRNA function during T-cell activation (Figure 2B). HMW-RISC complexes were associated with target repression and showed selective enrichment of certain miRNAs e.g., excluding miR-21 (74). It remains elusive how the assembly of HMW-RISCs is controlled.

## Conclusion

During the last decade, it has become evident that miRNAs play a critical role in development and function of Treg cells. The goal of gaining a better mechanistic understanding of miRNA function in these cells has driven technology toward “gold-standard” approaches to identify critically relevant one miRNA – one mRNA target relationships. At the same time, the case of miRNA-mediated control of Treg cells highlights the complexity of miRNA-mediated repression and the current lack of adequate technology to characterize quantitative aspects as well as miRNA – mRNA co-targeting networks.

## Author Contributions

HK-S and AK conceived, wrote, and edited the manuscript. Both authors contributed to the article and approved the submitted version.

## Conflict of Interest

The authors declare that the research was conducted in the absence of any commercial or financial relationships that could be construed as a potential conflict of interest.
